# Strategic Integration: A Cross-Disciplinary Review of the fNIRS-EEG Dual-Modality Imaging System for Delivering Multimodal Neuroimaging to Applications

**DOI:** 10.3390/brainsci14101022

**Published:** 2024-10-16

**Authors:** Jiafa Chen, Kaiwei Yu, Yifei Bi, Xing Ji, Dawei Zhang

**Affiliations:** Research Center of Optical Instrument and System, Ministry of Education and Shanghai Key Lab of Modern Optical System, University of Shanghai for Science and Technology, Shanghai 200093, China; 211180056@st.usst.edu.cn (J.C.); 223330689@st.usst.edu.cn (K.Y.); yifeibi@usst.edu.cn (Y.B.); 232200410@st.usst.edu.cn (X.J.)

**Keywords:** fNIRS, EEG, dual-mode monitoring, clinical application

## Abstract

**Background**: Recent years have seen a surge of interest in dual-modality imaging systems that integrate functional near-infrared spectroscopy (fNIRS) and electroencephalography (EEG) to probe brain function. This review aims to explore the advancements and clinical applications of this technology, emphasizing the synergistic integration of fNIRS and EEG. **Methods:** The review begins with a detailed examination of the fundamental principles and distinctive features of fNIRS and EEG techniques. It includes critical technical specifications, data-processing methodologies, and analysis techniques, alongside an exhaustive evaluation of 30 seminal studies that highlight the strengths and weaknesses of the fNIRS-EEG bimodal system. **Results:** The paper presents multiple case studies across various clinical domains—such as attention-deficit hyperactivity disorder, infantile spasms, depth of anesthesia, intelligence quotient estimation, and epilepsy—demonstrating the fNIRS-EEG system’s potential in uncovering disease mechanisms, evaluating treatment efficacy, and providing precise diagnostic options. Noteworthy research findings and pivotal breakthroughs further reinforce the developmental trajectory of this interdisciplinary field. **Conclusions:** The review addresses challenges and anticipates future directions for the fNIRS-EEG dual-modal imaging system, including improvements in hardware and software, enhanced system performance, cost reduction, real-time monitoring capabilities, and broader clinical applications. It offers researchers a comprehensive understanding of the field, highlighting the potential applications of fNIRS-EEG systems in neuroscience and clinical medicine.

## 1. Introduction

Neuroscience research is gaining increasing attention in the rapidly advancing field of modern medical technology. This heightened focus has elevated neuroimaging techniques to a pivotal role in the study of brain structure and function [1]. Among various neuroimaging methods, functional near-infrared spectroscopy (fNIRS) and electroencephalography (EEG) have emerged as primary tools for studying human brain activity [2,3]. The amalgamation of these two techniques into an fNIRS-EEG dual-modality imaging system holds the promise of surmounting individual technique limitations while substantially heightening neural signal-detection precision. Clinical neuroscience research and practice are expected to experience notable advancements in the coming years, opening up new possibilities for medical understanding and treatment [4].

**Figure 1:**
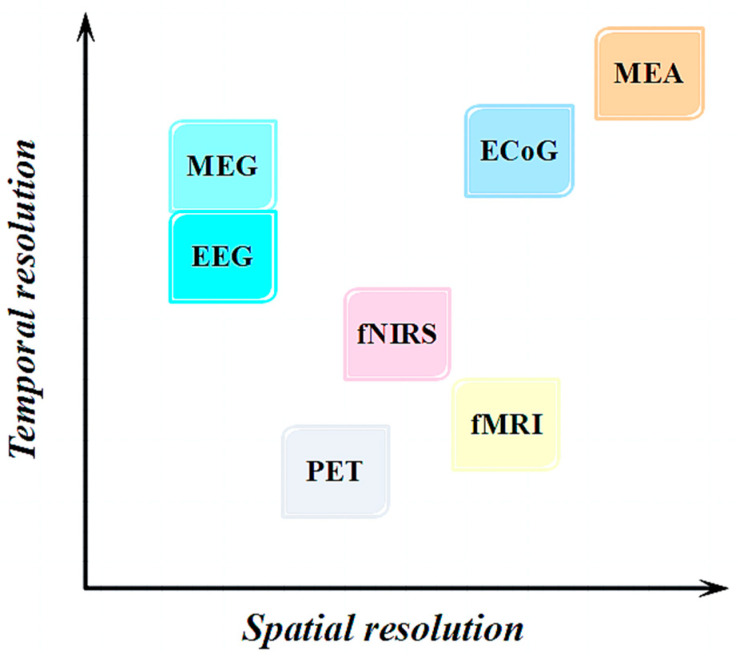
Comparison of the resolutions of commonly used functional brain-imaging techniques.

Brain science research methods must be selected according to specific research or clinical application requirements. This is illustrated in Figure 1. In terms of temporal resolution, the hierarchy of effectiveness, from highest to lowest, is as follows: microelectrode array (MEA), cortical electroencephalogram (ECoG), magnetoencephalography (MEG), electroencephalogram (EEG), functional near-infrared spectroscopy (fNIRS), functional magnetic resonance imaging (fMRI), and positron emission tomography (PET) [5]. Additionally, MEA provides the highest spatial resolution, followed by fMRI [6], EcoG, fNIRS, MEG and EEG offering the lowest spatial resolution. It is crucial to note that while both MEA and ECoG excel in temporal and spatial resolution, their measurements involve traumatic manipulation [7,8]. By contrast, MEG, fMRI, and PET necessitate costly equipment, require patient immobilization during testing, and, in the case of PET, entail the injection of a radiotracer [9]. Compared to these techniques, fNIRS captures signals related to blood-oxygen levels by monitoring changes in oxygenation (HbO) and deoxyhemoglobin (HbR) concentrations. This renders it non-invasive, compact, lightweight, and energy-efficient, establishing its widespread application in brain-function research [10].

EEG, based on neuronal electrical activity, detects brain activity by recording neurons’ spontaneous rhythmic movement potentials beneath the scalp. It automatically reflects an individual’s physiological and psychological changes, presenting real-time changes in brain activity. Consequently, it can extensively analyze and investigate various diseases and psychological states [11]. The acquisition process of EEG is straightforward and non-invasive, and it demonstrates commendable temporal resolution; this endows it with formidable capabilities in discerning brain activity [12]. Technically, fNIRS and EEG are complementary: EEG offers exceptional temporal resolution but relatively low spatial resolution, whereas fNIRS achieves notable spatial resolution due to the exponential attenuation of incident light in tissues [13]. Neither technique exhibits atypical physical constraints, and both can de-artifact. These techniques are particularly well-suited for tasks requiring more natural cognitive processes than fMRI, PET, or MEG. They can be applied to many individuals, from infants to the elderly. In addition, neither fNIRS nor EEG require high levels of magnetic field or ionizing radiation, and their hardware costs are significantly lower than many other functional brain-imaging methods.

In recognition of these technical strengths and limitations, researchers have explored a dual-modality imaging system that integrates fNIRS and EEG. Such a system would surmount the limitations inherent in single-modality functional brain analyses while providing insights into cortical electrical activity and metabolic hemodynamics without electromagnetic interference. It is, therefore, ideal for applications in non-laboratory settings, such as natural environments, portable monitoring setups, and bedside settings [14]. Neurological disorders have increased significantly in response to the escalating trend of population aging [15]. It is, therefore, imperative to utilize precise neuroimaging techniques in this context since they can provide valuable information for early diagnosis, ongoing disease monitoring, and treatment evaluation.

This review provides a comprehensive overview of research on fNIRS-EEG dual-modality imaging systems in clinical applications. We begin by elucidating the fundamental principles and characteristics of fNIRS and EEG technologies, highlighting their pivotal roles in neuroimaging. We then examine the integration of fNIRS and EEG within dual-modality systems, focusing on the fusion modalities and exploring their diverse applications in clinical neuroscience. These applications include attention-deficit hyperactivity disorder (ADHD), infantile spasms, depth of anesthesia estimation, intelligence quotient (IQ) assessment, and epilepsy. Our synthesis of relevant research findings underscores the significant contributions of fNIRS-EEG dual-modality imaging in enhancing diagnostic precision, enabling tailored treatment strategies, and advancing scientific inquiry. Additionally, we provide an overview of the characteristics, limitations, and methodologies used for data processing and analysis within the fNIRS-EEG framework across various application areas. As we traverse the existing body of research, we also assess future trends in fNIRS-EEG dual-modality imaging systems. Our aim is to emphasize the importance of technological innovation and the necessity for interdisciplinary collaboration to address technical challenges. Such investigations are crucial for deepening our understanding of the brain and its associated disorders.

## 2. fNIRS-EEG Dual-Modality Imaging System, Fusion-Detection Approach

**Figure 2:**
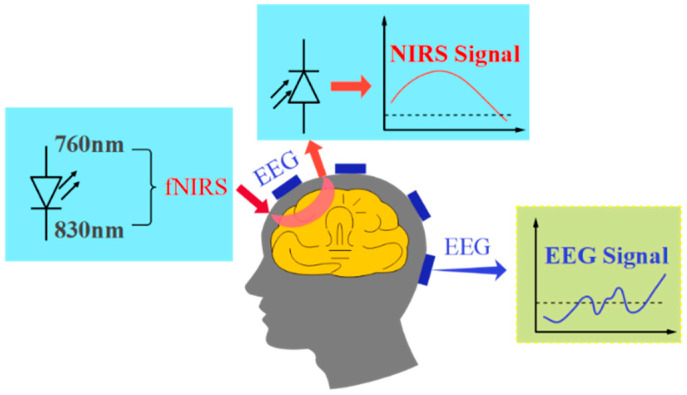
fNIRS-EEG dual-modality imaging system fusion method for assessing brain functions.

As depicted in Figure 2, fNIRS and EEG techniques require precise scalp localization to acquire the signals essential for a comprehensive evaluation of functional brain activity [16]. A fusion-detection system is typically comprised of EEG electrodes, fNIR probes, a lower computer microcontroller, and upper computer software. The quantity and distribution of EEG electrodes and fNIR probes must be meticulously configured to meet specific requirements. The microcontroller serves as the central component of the system. It generates drive signals for the light source, amplifying intensity signals from fNIRS and EEG. It conducts analog-to-digital conversion and establishes communication with the host computer. Conversely, the host computer has responsibilities encompassing preprocessing, fusion analysis, mathematical modeling, and the facilitation of human–computer interaction with the fNIRS and EEG signals [17].

Generally, there are two methods for integrating fNIRS and EEG signals. As demonstrated by Fazli et al., a combination of fNIRS and EEG data, obtained separately using the NIRScout and BrainAMP systems and synchronized for acquisition and analysis via a host computer, improved real-time EEG classification accuracy [18]. Despite the relative simplicity of this approach, it is essential to note that the synchronization may not achieve the level of precision required for the analysis of EEG data with microsecond time resolution.

Meanwhile, Ge et al. utilized a unified processor simultaneously to process and acquire EEG signals together with fNIRS input and output, resulting in relatively high accuracy [19]. Although this method requires a more complex and intricate system design, it achieves precise synchronization between the two systems, streamlining the analytical process. Currently, this method is the most widely used approach for concurrent fNIRS and EEG detection.

### Helmet Fusion for Fnirs-EEG Dual-Modality Imaging-System Acquisition

The joint-acquisition helmet design holds paramount importance. Currently, in the fusion detection of fNIRS and EEG, the NIR probe and EEG electrodes are commonly integrated into the same acquisition helmet [20]. Specific design approaches include integrating the EEG electrodes and NIR probes on a shared substrate material or arranging the EEG electrodes separately from the NIR fiber-optic components. In the latter approach, the spatial arrangement of the EEG electrodes assists in co-registering the EEG and fNIRS channels, enabling precise spatial localization of the brain regions probed by the NIR measurement channels. This co-registration is essential for accurate spatial alignment between the two modalities, facilitating a more integrated interpretation of the electrophysiological and hemodynamic data [21]. Nevertheless, some researchers have directly integrated NIR fiber optics into existing EEG electrode caps [22]. While both methods are relatively straightforward to implement and maintain a satisfactory EEG-fNIRS coupling, they present two primary challenges. First, prevailing EEG electrode caps frequently employ elastic fabric as a substrate. This may result in uncontrollable variations in the distance between the NIR light source and the detector when the apparatus is worn by different subjects. (This stems from variations in head shapes.) Second, the effectiveness of elastic fabric in securing the NIR probe is limited due to its high stretchability, which may lead to inconsistencies in probe placement and variations in the distance between the fNIRS light source and detectors. These inconsistencies can result in fluctuating probe-to-scalp contact pressure, especially during movement or long-duration experiments, which negatively impacts data quality and the accuracy of the experimental results. Furthermore, these challenges are closely related to the movement artifacts and scalp-coupling variability discussed later in the manuscript.

To address this issue, researchers have turned to 3D printing technology to craft customized joint-acquisition helmets tailored to experimental requirements [23]. This innovative helmet design allows for the flexible positioning of EEG electrodes and NIR probes, accommodating head-size variations among subjects. While this method has demonstrated promising results in experiments, one should note that 3D printing costs are relatively high. Additionally, researchers have explored the use of a composite polymer cryogenic thermoplastic sheet for crafting joint-acquisition helmets. This material, widely utilized in clinical orthopedic and radiotherapy physics centers to immobilize patients, can be softened and shaped at around 60 °C, retaining its form stability upon cooling. This attribute makes cryogenic thermoplastic sheets an excellent choice for customized helmet construction, with the base readily fashioned following the measurement site [24]. Still, despite its advantages of being cost-effective and lightweight, there are potential issues to consider, such as the possibility of the molded polyethylene thermoplastic sheet being slightly rigid and exerting some pressure when worn on the head.

At present, a common approach among researchers involves the utilization of flexible EEG electrode caps as a foundation for integrating both EEG and fNIRS technologies. Typically, punctures are made at specific locations to accommodate the fNIRS probe fixtures, and plastic connectors are used to maintain probe spacing [25]. Some designs also incorporate structures that help secure the probes while ensuring wearer comfort and reducing costs. These setups, as described in previous research, offer a practical solution for combining bimodal imaging techniques within a single cap configuration [25]. This method has proven cost-effective and comfortable for wearers. Nonetheless, it is imperative to exercise caution to prevent potential conflicts between the positions of the EEG electrodes and NIR probes during spatial coupling. In designing a joint-acquisition helmet, it is crucial to ensure not only the efficacy of the collar for EEG electrodes but also the steadfast fixation between the NIR light source and the detector.

## 3. fNIRS-EEG Dual-Modality Imaging System in Clinical Practice

The fNIRS-EEG dual-modality imaging system has emerged as an indispensable tool across many research and application domains. This paper comprehensively explores its significant contributions in these realms, specifically by categorizing its application areas in terms of five key themes. Through in-depth discussions of current research focal points, we aim to offer a thorough understanding of the far-reaching impact of fNIRS-EEG technology in the fields in question. The five themes illuminate the profound influence of fNIRS-EEG technology across diverse research and application landscapes, offering fresh perspectives and innovative solutions for a spectrum of disciplines. In the subsequent subsections, we will expound upon the pivotal aspects of each topic, presenting a more nuanced view of the extensive applications of fNIRS-EEG technology. Through these detailed discussions, we aim to provide a glimpse into certain intriguing facets of this technology and the significant achievements it has facilitated in the realms of science, medicine, and technology.

### 3.1. ADHD

**Figure 3:**
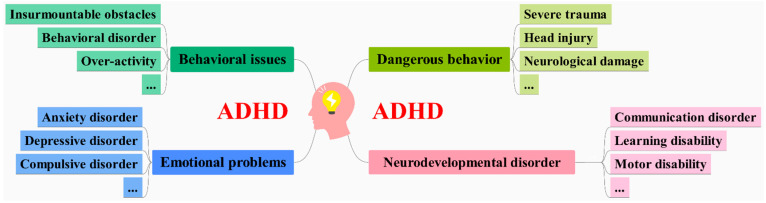
Accompanying symptoms of ADHD in children and adolescents.

ADHD is a prevalent neurodevelopmental condition characterized by age-inappropriate and persistent traits of inattention, hyperactivity, and impulsive behavior. It is notably commonplace among school-age individuals, with a global incidence of 7.2% [26] and a specific rate of 6.26% in China [27]. These ADHD symptoms tend to endure over time, and they are often accompanied by additional manifestations, encompassing neurodevelopmental deficits, emotional challenges, behavioral issues, and potentially risky behaviors, as illustrated in Figure 3. Diagnoses of ADHD rely predominantly on assessments conducted through interviews with teachers or parents, rooted in the criteria outlined in the DSM-IV or DSM-V [28]. Nevertheless, this diagnostic methodology primarily hinges on behavioral evaluations, lacking robust physiological data support. This gap in objective measurement poses challenges in accurately discerning the severity of the disorder. Consequently, in recent years, there has been a proposal to employ fNIRS-EEG dual-modality imaging to enhance the precision of ADHD diagnosis. By garnering more comprehensive physiological data and refining detection accuracy, this approach evinces potential for early ADHD diagnosis [29].

Recent advancements in neuroimaging and physiological research methods have introduced novel avenues for assessing hemodynamic changes within the prefrontal cortex (PFC) in children with ADHD [30,31]. These investigations have revealed that most children diagnosed with ADHD exhibit deficits in prefrontal activity [32]. These deficits manifest in various brain regions, notably impacting the anterior cingulate cortex, dorsolateral prefrontal cortex, and supplementary motor areas, with pronounced differences apparent during inhibitory control tasks. Comparatively, when contrasted with their neurotypical peers, children with ADHD display functional abnormalities in several neural networks, including the default network, ventral/dorsal attentional network, salience network, frontal striatum, and midbrain cortical and limbic circuits [33,34,35]. These aberrations hold the potential to serve as objective biomarkers in the diagnosis of ADHD [36].

In clinical research on ADHD, fNIRS has been employed to monitor alterations in relative HbO, HbR, and total hemoglobin (Hb) levels across different task states. Given the heightened sensitivity and reliability of HbO to changes, researchers have placed greater emphasis on tracking variations in HbO concentrations. Study results indicated that, during cognitive tasks, the functional activity of the PFC in children with ADHD tended to decrease. However, HbO levels in the ADHD group increased compared to the control group, reflecting a compensatory hemodynamic response. This neurovascular uncoupling, where increased HbO is not necessarily indicative of heightened neural activity, is commonly observed in ADHD populations [37]. Furthermore, this phenomenon was accompanied by cognitive deficits in areas such as verbal fluency, sustained performance tests, the Stroop task, the proceeding/non-proceeding paradigm, and working memory [38,39]. EEG measurements have been employed to assess differences between children with ADHD and their counterparts in the control group by analyzing resting-state and task-related α, β, and θ power, as well as event-related N2 and P3 components [40]. The study outcomes revealed elevated relative theta power and reduced relative alpha and beta activity, along with elevated theta/alpha and theta/beta ratios in children with ADHD compared to controls. These findings are most reliably associated with ADHD and may indicate different profiles of cortical anomalies [41]. This indicates that children with ADHD demonstrate lower complexity levels than their healthy counterparts. The fNIRS-EEG technique, a non-invasive tool, holds considerable significance in gauging the extent of impairment in children with ADHD. It presents a novel avenue for identifying biomarkers and facilitating early diagnosis and treatment.

**Figure 4:**
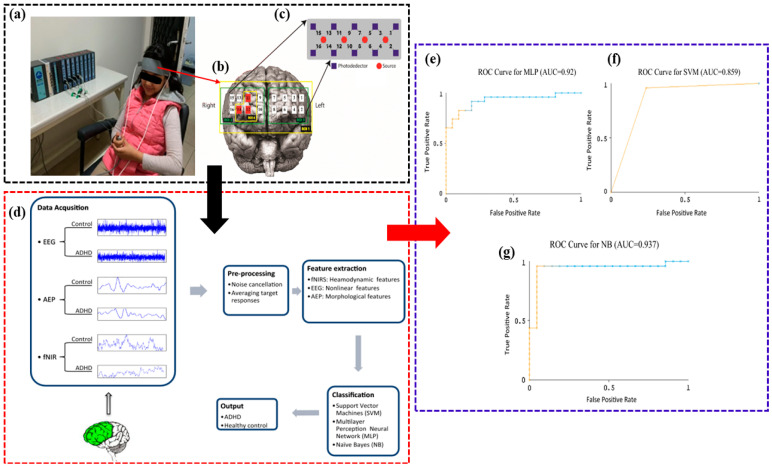
Visualization of the fNIRS-EEG dual-modality imaging system for diagnosing children with ADHD [29]. (**a**) EEG electrodes were placed on the Fz (frontal), Pz (parietal), Oz (occipital) and Cz (central) locations according to the international 10–20 system. A band with optical fiber probes was placed on the forehead for fNIRS data acqusition. Data acquisition system has been shown on a control subject. (**b**) Spatial profiles of the fNIRS channels and the ROIs locations. (**c**) The source-detector and 16 optode (channel) measurement locations registered on fNIR probe. (**d**) The flowchart of the study (the signals belong to a random control and ADHD subject). (**e**) ROC Curve for MLP (AUC = 0.92). (**f**) ROC Curve for SVM (AUC = 0.859). (**g**) ROC Curve for NB (AUC = 0.937).

The study by Güven et al. introduced a new method to distinguish children with ADHD from healthy controls using a simultaneous fNIRS-EEG dual-modality imaging system (fNIR Imager 1100) [29]. EEG electrodes were placed according to the International 10–20 System, as shown in Figure 4. The study involved 23 children with ADHD and 21 healthy children. The results indicated that compared to the control group, children with ADHD had lower complexity values, longer P3 latency, and reduced P3 amplitude. Additionally, fNIRS data showed that healthy children exhibited higher right prefrontal activation. The researchers applied various machine learning techniques, including Naïve Bayes, which achieved the highest classification accuracy of 79.54% using EEG alone and 93.18% using the combined EEG-fNIRS system. These findings suggest that combining fNIRS and EEG data improves the accuracy of ADHD diagnosis.

### 3.2. IQ Estimation

IQ is a pivotal metric for gauging an individual’s cognitive prowess, significantly influencing their learning capacity, cognitive abilities, and overall achievements [42]. Consequently, precise estimation of IQ holds paramount importance for educational assessments, cognitive studies, and the diagnosis and treatment of intellectual disabilities [43].

Conventional approaches to IQ assessment primarily rely on psychometric tests, such as the Wechsler Intelligence Scale. However, these methodologies face limitations in certain situations, particularly when applied to children or individuals with cognitive disabilities who may be unable to fully participate in the required psychological tasks. Additionally, many neuroimaging methods used to assess cognitive function also require some level of cognitive engagement, which can further restrict their applicability in these populations.

In previous research, EEG has been employed to delineate the impact of varying IQ levels on the resolution of diverse mathematical problems, ultimately for IQ estimation purposes [44]. These findings indicated a negative correlation between IQ and cerebral activity in specific cognitive tasks or conditions [45], with the brain demonstrating consistent, self-similar EEG patterns during cognitively relevant tasks [46]. Nevertheless, there are certain limitations to this approach. For instance, the connection between task-related events and cognitive or mental activities lacks uniqueness and correspondence. Furthermore, motion artifacts induced by eye movements, respiration, and other factors can further compromise the stability and precision of EEG signals.

On the other hand, fMRI can discern pertinent cerebral events during other types of cognitive tasks, where IQ often exhibits a positive correlation with brain activity [47]. Nevertheless, this technique has constraints, including lower temporal resolution, elevated expenses, lack of portability, and relatively intricate operation.

Researchers conducted an in-depth analysis of brain network functional connectivity in children during rest and language tasks using fNIRS-EEG dual-modality imaging technology. Based on the functional connectivity matrix derived from changes in fNIRS oxygenated hemoglobin concentration, the results indicate that connectivity in the left hemisphere of children with epilepsy is significantly weakened, while local connectivity in the right hemisphere is enhanced. Compared to healthy controls, children with epilepsy exhibited significantly poorer cognitive performance, and the local network efficiency in the left hemisphere showed a negative correlation with IQ. This suggests that although epilepsy leads to brain reorganization, this reorganization does not facilitate optimal cognitive development [48].

**Figure 5:**
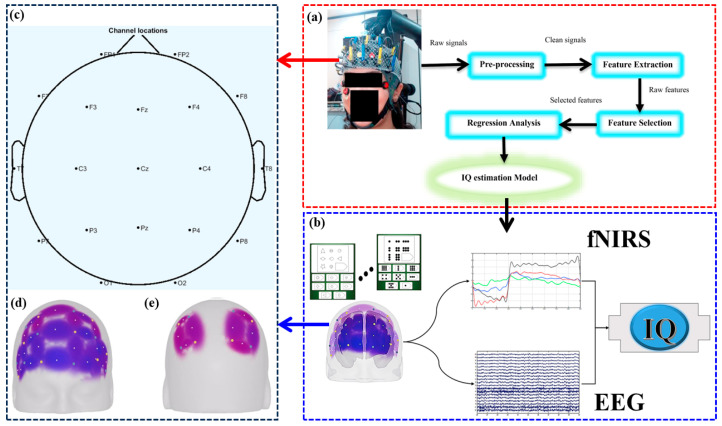
Visualization of the fNIRS-EEG dual-modality imaging system for studying IQ estimation [49]. (**a**) The overall scheme of the IQ estimation procedure. (**b**) IQ Experiment Flowchart. (**c**) the location of EEG electrodes. (**d**) fNIRS Optodes placement (**e**) channel configuration for problem solving task.

Firooz et al. employed a bimodal fNIRS-EEG dual-modality imaging system to evaluate an individual’s IQ during the Raven’s Progressive Matrix (RPM) intelligence test, as illustrated in Figure 5 [49]. Their study highlights the potential of this approach in assessing not only intellectual development and cognitive dysfunction in clinical settings but also in educational assessments and individual developmental studies. By utilizing fNIRS and EEG, they aimed to estimate IQ without requiring a complete logical-mathematical intelligence test, demonstrating a broad spectrum of applications for fNIRS-EEG dual-modality imaging in understanding individual differences in intelligence.

### 3.3. Infantile Spasm

Infantile spasms constitute a prevalent neurodevelopmental disorder characterized by transient and rapid muscle contractions, primarily occurring during the early stages of a child’s growth [50]. While the precise etiology and pathogenesis of infantile spasms remain only partially understood, extensive research suggests the involvement of various neuronal pathways and signaling irregularities [50,51]. Presently, clinical observations indicate that infantile spasms entail particular physiological processes that are both age-dependent and centered around the subcortical pathways [52]. Traditional techniques, such as PET and single-photon emission computed tomography (SPECT), offer insights into cortical malformations’ hemodynamic and metabolic aspects, distal cortex, subcortical structures, and the brainstem [53]. Additionally, activity within cortical malformations can stimulate neuronal networks in the brainstem, potentially contributing to spasticity. Given that an understanding of the pathophysiological mechanisms associated with spasticity demands exceptionally high temporal resolution, conventional monitoring tools have struggled to meet clinical demands. As a result, researchers have turned to fNIRS-EEG technology to monitor brain function in infantile-spasticity patients, presenting a potential breakthrough in diagnosis and treatment. This approach also offers robust support for exploring new therapeutic modalities and intervention strategies.

In clinical studies of infantile spasms, fNIRS has proven to be a valuable tool for monitoring successive hemodynamic events that occur during spasms [54]. This technology allows researchers to differentiate between changes in blood volume related to vaso-regulation and those associated with neurovascular coupling, which is indicative of neuronal activation [55]. Investigations revealed a biphasic pattern of hemodynamic changes during spasticity. The initial phase primarily engages the brainstem nervous system, potentially influencing cerebral blood flow (CBV) regulation by modulating the contractility of intracerebral blood vessels in the peripheral and deeper regions of the brain [56]. This leads to swift and concurrent alterations in the concentrations of HbO and Hb. Moreover, the activation of brainstem structures sets off spastic pattern generators akin to reflexes such as the Moro reflex, startle response, sudden sneezing, and coughing. This activation also initiates synchronized motor clusters independent of cortical circuits, influencing changes in HbO and Hb concentrations. The second phase manifests within 6 to 10 s following the onset of spasticity. Brainstem intervention prompts heightened HbO concentrations and diminished HbR concentrations. The role of neurovascular coupling in infantile spasms aligns closely with the classical balloon model. EEG patterns demonstrate the characteristic involvement of specific subcortical and cortical processes and structures in clinical and electroencephalographic phenotypes. Secondary diffuse activation of the brainstem may lead to widespread changes in cortical excitability, resulting in diffuse high-pressure slow waves [57]. These waves correlate with background activity and fast rhythm alterations, as observed in the EEG [58].

**Figure 6:**
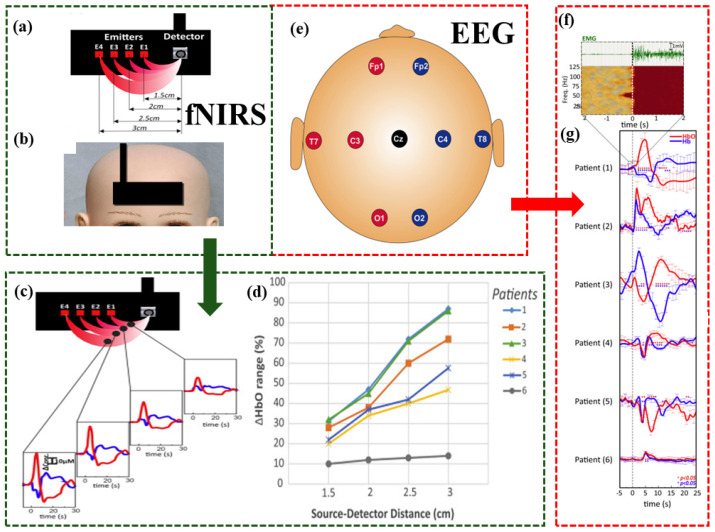
Visualization of the fNIRS-EEG dual-modality imaging system for studying infantile spasms [57]. (**a**) fNIRS: a patch composed of four pairs of optical fibers (each wavelength corresponds to one thread in each team), containing four transmitters and one detector. (**b**) fNIRS: a detector has been positioned in the middle of the forehead. (**c**) fNIRS: Hemodynamics observed over multiple distances via fNIRS spectroscopic technique graphs. (**d**) fNIRS: Normalized range values of [HbO] for the four source-detector distances in the period of −5 to 25 s vs source-detector distances (1.5, 2, 2.5, 3 cm) for the 6 patients. (**e**) EEG: layout of nine electrodes (10–20 system configurations, with a frontal reference). (**f**) A time-frequency response (TFR) of the deltoid EMG determined the onset of each infantile spasms (T0). infantile spasms onset was always characterized by a sudden increase in the deltoid EMG power of all frequency bands between 0 and 100 Hz. (**g**) A two-phase hemodynamic change started with the onset of EMG activation (as determined in a time-frequency analysis).

The study by Bourel-Ponchel et al. [57] indicates that infantile spasms are a complex epileptic manifestation involving both cortical and subcortical structures, as illustrated in Figure 6. The existence of diffuse cortical neurovascular coupling (NVC) associated with increased brain metabolism during each spasm reflects widespread cortical activation. However, NVC was not observed in cases with large porencephalic cysts, suggesting that the cortex may not be involved in all infantile spasms. These findings also indicate that the NVC was preceded by diffuse changes in cerebral blood volume (CBV), implying possible initial involvement of the brainstem. By using simultaneous EEG-fNIRS monitoring, researchers can further explore the complex dynamic interplay between brain activity, blood volume, and metabolism during infantile spasms.

### 3.4. Epilepsy

Epilepsy encompasses a group of prevalent and chronic neurological disorders characterized by epileptic seizures, also called ictal episodes, which are triggered by abnormal and excessive neuronal discharges [59]. These seizures typically range from a few seconds to a few minutes. They are often accompanied by altered mental status, tonic and clonic movements, and other perceptual-experiential symptoms [60]. In the intervals between these seizure events, there may be brief bursts of discharges lasting between 20 and 200 milliseconds, commonly known as “interictal epileptiform discharges” (IEDs, or simply “spikes”).

Early studies primarily relied on EEG/fMRI for the clinical assessment of epilepsy. This non-invasive method involved the monitoring of spike activity in cortical areas during seizures [61]. Nevertheless, both approaches have their respective limitations. The EEG single modality suffers from low spatial resolution, which poses challenges in accurately pinpointing seizure regions. On the other hand, fMRI is not applicable during the unpredictable interictal period, and it can be affected by behavioral changes (such as violent head and body movements) during seizures.

Furthermore, certain studies have employed SPECT or PET to track cumulative hemodynamic changes throughout seizures [62]. Nonetheless, the efficacy of these two methods could be more robust in their application. This is primarily attributed to poorer temporal resolution, which hinders the differentiation of ipsilateral hemodynamic changes during the seizure. Additionally, the influence of seizure propagation patterns on hemodynamic changes and the quantifying of cerebral blood flow during a seizure remains an area in need of improvement.

Several researchers have observed that heightened neuronal activity during seizures subsequently influences brain metabolism and cerebral blood flow, resulting in alterations in hemodynamic and oxygenation profiles [63,64]. Recognizing the limitations of fMRI and PET/SPECT in capturing these dynamics, certain scholars have pioneered the application of the fNIRS-EEG dual-modality imaging technique to the preoperative assessment of patients with medial temporal lobe epilepsy. More specifically, they have utilized it as a diagnostic adjunct for the prolonged monitoring of various types of epilepsy [65].

The fNIRS-EEG technique holds a pivotal role in clinical applications concerning epilepsy. Through fNIRS, one can acquire indicators regarding hemodynamic changes, including alterations in the concentration of HbO and HbR and mean blood-flow velocity. Study findings demonstrated a localized increase in oxygen–hemoglobin signal within the seizure region for most subjects [64]. EEG proved effective in identifying five distinct events characterized by clinically symptomatic definitions, encompassing EEG activity suppression, spike sequences, theta bursts, clinical seizures, and aura [66]. Meanwhile, the notable correlation between various EEG patterns and hemodynamic changes furnishes a vital foundation for a profound exploration and more robust comprehension of the functional state of the brain in patients with epilepsy.

**Figure 7:**
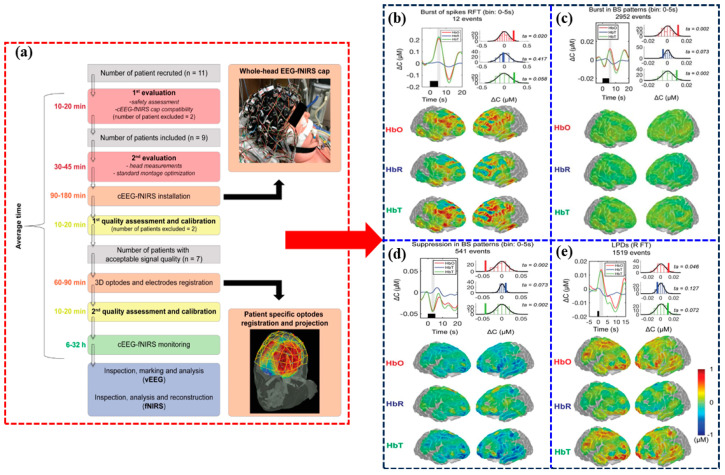
Visualization of the fNIRS-EEG dual-modality imaging system for the study of epilepsy [67]. (**a**) Sketch plot of the patient evaluation, continuous encephalography and fNIRS data acquisition, and data analysis procedures (**b**) Hemodynamic changes associated with nonconvulsive seizures. (**c**) Hemodynamic changes associated with BS bursts. (**d**) Hemodynamic changes associated with burst suppression suppressions. (**e**) Hemodynamic changes associated with periodic discharges.

Kassab et al. introduced a methodology that employed the fNIRS-EEG dual-modality imaging system to monitor cortical hemodynamics in conjunction with status epilepticus, burst suppression, and periodic discharges [67], as illustrated in Figure 7. The findings of this study indicated that during seizures, bursts, and frequent eruptions, fNIRS activity exhibited a general increase in HbO alongside a decrease in HbR within the brain. The magnitude and duration of these hemodynamic changes positively correlated with the length of the event observed in EEG. This preliminary research showcased the viability of employing the fNIRS-EEG dual-modality imaging system within a neuro-intensive care unit.

### 3.5. Precise Monitoring of the Depth of Anesthesia

In recent years, the monitoring of anesthesia depth has assumed an increasingly critical role in clinical practice, evincing paramount importance for the safety of patient surgery and subsequent postoperative recovery [68]. Research has underscored that inadequate monitoring can result in issues such as confusion, pain, and anxiety during surgery, potentially leading to post-traumatic stress disorder, depression, and/or sleep disorders. Conversely, excessive monitoring may give rise to neurological complications, sleep apnea, and cardiovascular issues [69]. Therefore, precise neurological status monitoring is pivotal in elevating the quality of surgery and enhancing patients’ overall quality of life.

Currently, methods for assessing the depth of anesthesia can broadly be categorized into two main approaches. The first relies on the subjective observation of clinical indicators by medical personnel, including heart rate, blood pressure, and blood-oxygen levels [70]. The second method involves the employment of EEG-based metrics, such as the Bispectral Index (BIS) monitor and entropy, for the continuous monitoring of the frontal lobe [71]. Nevertheless, EEG exhibits several limitations in clinical applications, such as susceptibility to interference from other high-frequency noises, specificity for anesthetic drug-induced response changes, and a constrained application environment for the associated equipment. Conventional methods must be revised to meet clinical demands for high reliability, accuracy, multimodality, and portability. Given the range of drug-type-specific neural and cerebral blood-flow changes induced by anesthesia, numerous studies have turned to fNIRS to assess neurovascular coupling and capture neuronal signals. This approach characterizes depth of anesthesia with heightened sensitivity, utilizing changes in HbO and Hb concentrations. This, in turn, furnishes a richer set of information regarding the depth of anesthesia [72].

**Figure 8:**
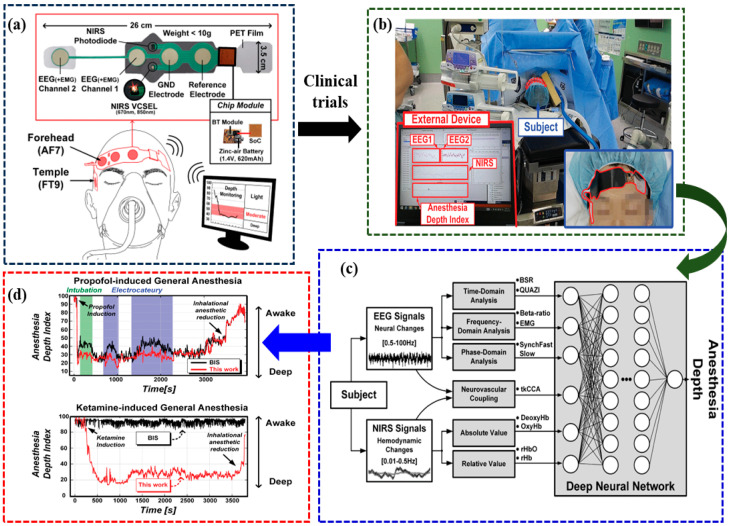
Visualization of the fNIRS-EEG dual-modality imaging system for studying depth of anesthesia [69]. (**a**) Proposed multimodal anesthesia depth monitoring system. (**b**) Clinical trial environment. (**c**) Flowchart of the deep-learning algorithm. (**d**) Clinical results for propofol-induced general anes-thesia and ketamine-induced general anesthesia.

Ha et al. introduced a lightweight, wearable fNIRS-EEG dual-modality imaging integrated system, utilizing 65 nm CMOS technology and enabling non-invasive and dependable anesthesia depth monitoring [69], as illustrated in Figure 8. This system concurrently tracks a patient’s cerebral blood-oxygen levels through a near-infrared light sensor, and it records EEG signals to observe EEG activity. Subsequently, it integrates these two datasets to offer a more precise assessment of a patient’s depth of anesthesia. Given the demonstrated effectiveness of this approach, it holds promise for potential clinical applications within the realm of anesthesia monitoring and management.

## 4. Literature Summary for fNIRS-EEG Dual-Modality Imaging-System Applications

We conducted a detailed quantitative analysis of the existing fNIRS-EEG dual-modality imaging systems to reveal their key features and application scenarios. The following presents the key information extracted from the attached Tables S1–S3:

### 4.1. Comparison of System Characteristics

According to the attached Table S1, the main characteristics of the systems include the number of channels, sampling rate, and optical wavelength, which significantly impact system performance and application outcomes:

- Number of channels: The number of optical channels in fNIRS systems varies depending on specific research needs. For instance, Kassab et al.’s [67] system has 128 optical channels, while He et al.’s [73] system has 36 channels. A higher number of optical channels supports broader brain region coverage, especially for complex tasks like bimanual training tasks [74]. In contrast, Liu et al.’s [75] system has only 16 optical channels, which is more suited for simpler tasks such as working memory tasks. Changes in detector spacing may affect signal depth detection. For instance, Yi et al.’s [76] system has a detector spacing of 35 mm, suitable for deeper brain region monitoring, while Chiarelli et al.’s [77] system uses a 15 mm spacing, better suited for shallow region monitoring. Furthermore, there are notable differences in the number of EEG channels across systems. For example, Sun et al.’s [78] system has 14 EEG channels, while Xu et al.’s [79] system has 64 channels, offering greater potential for higher decoding accuracy in brain–computer interface tasks like motor imagery. A higher number of channels provides better spatial resolution for EEG signals, particularly in studies involving complex brain functions.
- The sampling rates of fNIRS and EEG also vary between systems. For example, the system by Ortega et al. [80] has an fNIRS sampling rate of 12.5 Hz, while the EEG sampling rate reaches 4000 Hz, making it suitable for tasks requiring high temporal resolution. The system by Yi et al. [76] has an fNIRS sampling rate of 10 Hz and an EEG rate of 500 Hz, allowing for the simultaneous acquisition of hemodynamic and electrophysiological signals.

### 4.2. Application Scenario Analysis

The attached Table S2 compares the performance of various systems in practical application scenarios:

- In practical applications, the portability and wearability of the system directly affect its potential for widespread use. The systems by Chu et al. [81] and Aghajani et al. [82] performed poorly in terms of portability, with equipment that is often bulky and not suitable for long-term wear or large-scale brain region monitoring. Although some systems still lack portability and wearability, newer systems have introduced wireless operations and lightweight designs [69], improving portability.
- Significant differences exist in data acquisition accuracy and processing capabilities across systems. Early systems [78,83] validated the concept of multimodal integration, but their data processing accuracy was limited due to low fNIRS sampling rates and restricted brain region coverage. Current systems have improved acquisition accuracy, such as the full-brain measurements in Yi et al. [76], but still face issues such as low sampling rates and limited data volume. Additionally, some studies [73,84] have enhanced classification performance through data augmentation and feature fusion techniques, demonstrating the potential for improving model quality.

### 4.3. Comprehensive Evaluation

Through a detailed comparison of these quantitative data, we can assess the practical performance and applicability of different fNIRS-EEG dual-modality imaging systems more accurately. These data provide an objective foundation for understanding the strengths and weaknesses of existing technologies, guiding the future development of equipment. For example, balancing system comfort with the number of channels and data processing capabilities, as well as balancing portability with full-brain measurement capability, are key directions for future improvements.

## 5. Challenges and Future Trends in fNIRS-EEG Dual-Modality Imaging Systems

### 5.1. Hardware Issues

The primary hardware issues focus on wearing comfort, device stability, and multitasking capabilities. Although the current system has improved comfort compared to other systems, discomfort still occurs during long experiments, especially when participants engage in complex cognitive tasks. Head movement and perspiration can affect sensor stability, reducing data quality. To address this, future research should focus on developing lighter and more breathable headgear, utilizing flexible sensors to enhance comfort and fit. Similar to Jiang et al.’s [74] whole-brain measurement system, while it performs well in hardware complexity, its portability and ease of wearing still need improvement. This suggests that future research should focus on device miniaturization and breakthroughs in wireless transmission technology. Reducing the number of external cables and optimizing sensor placement can significantly improve the device’s user experience.

Significant challenges remain in the current hardware design regarding data synchronization and multimodal integration. fNIRS and EEG signals are collected by different devices, relying on optical sensors and electrode arrays, respectively, which can cause transmission delays. While Su et al.‘s [85] system offers significant multitasking advantages, there are still potential errors in complex task signal synchronization. Thus, future designs need to incorporate more advanced synchronization algorithms and employ high-bandwidth, low-latency wireless transmission protocols (such as 5G and Wi-Fi 6) to reduce such errors. Further hardware integration will enhance the synchronization accuracy of dual-modal signals, improving overall system stability and performance.

### 5.2. Software Issues

Software issues are also one of the main challenges faced by fNIRS-EEG systems, particularly in data processing and analysis. Current analysis processes often require multiple preprocessing steps, including denoising, filtering, and artifact removal, which increases system complexity. Ortega et al.’s [80] system shows excellent performance in data classification, but confusion remains during decoding, especially with suboptimal multimodal data fusion in complex tasks. This indicates a need for more efficient algorithms to handle data preprocessing and feature extraction. Future algorithms should evolve toward automation and intelligence. Deep learning and reinforcement learning models can automatically extract meaningful features from large fNIRS and EEG datasets, reducing reliance on manual feature selection. Although some systems [86] use simpler preprocessing methods, more complex signal-processing algorithms, such as adaptive filtering or convolutional neural networks, can be introduced to improve data processing efficiency and classification accuracy in the future.

Another software challenge is the standardization and sharing of data across laboratories. Differences in equipment, data processing software, and analysis procedures across research institutions lead to inconsistent data formats and difficulties in data sharing. To promote collaboration between studies, future research should establish a standardized fNIRS-EEG data format. Similar to standardized protocols in fMRI (such as BIDS), developing a unified data sharing and processing framework can significantly improve data comparability and reproducibility. Additionally, cloud-based processing platforms will allow multiple laboratories to perform collaborative analysis on shared data, enhancing research efficiency.

### 5.3. Future Trends

The development of fNIRS-EEG multimodal systems will progress toward improving system usability and expanding application scenarios. Currently, these systems are primarily used in laboratory settings, but the future goal is to transform them into portable, wearable devices that can monitor brain activity in real time during daily life. Therefore, future fNIRS-EEG systems should focus more on portability and ease of wear, such as developing sensor devices that can be integrated into hats or headbands and extending applications to non-laboratory settings like homes or hospitals for real-time brain function monitoring.

With the introduction of artificial intelligence technology, real-time data analysis and feedback will become key development directions for future fNIRS-EEG systems. For example, while Hamann et al.’s [84] system excels in data augmentation and multimodal signal fusion, its small sample size limits its broader application. Future systems should automatically detect and process noise signals, generate real-time analysis reports, and assist users in making quick decisions in clinical or rehabilitation processes. Meanwhile, real-time monitoring will require devices to have greater computing power and battery life, which will be a key focus of future research.

Multimodal data fusion is an important direction for future research, especially in areas such as epileptic seizure detection, where both fNIRS and EEG are valuable [87,88]. EEG detects abnormal electrical activity during seizures [89], while fNIRS complements this by capturing cerebral oxygenation changes [90,91]. Although current systems can simultaneously collect fNIRS and EEG signals [92], a better fusion of these modalities is needed to provide more comprehensive brain function information. For example, Xu et al. [79] made significant progress in feature fusion methods, but the limited number of channels still constrains fusion accuracy. Future research should develop more advanced multimodal fusion algorithms, leveraging machine learning techniques [91] to fully utilize the complementarity of fNIRS and EEG signals, improving brain signal decoding accuracy in complex tasks. This will not only advance research on cognitive functions but also promote the development of BCIs, including hybrid fNIRS-EEG systems, which have shown improved accuracy in motor imagery decoding [93], with applications in neurofeedback therapy, where real-time feedback on electrical and hemodynamic signals can aid in the self-regulation of brain activity for conditions like ADHD and epilepsy [94], cognitive rehabilitation [95], and even smart device control.

In conclusion, the future development of fNIRS-EEG multimodal systems will focus on hardware miniaturization, intelligent software algorithms, multimodal data fusion and standardization, and cross-context application expansion. By overcoming current challenges, future fNIRS-EEG systems will play a greater role in clinical, research, and daily life settings, driving the advancement of brain science and neurotechnology.

## 6. Conclusions

This study comprehensively examines the clinical applications of fNIRS-EEG dual-modality imaging systems across various fields while addressing associated challenges and prospects. Initially, we elucidated the principles and attributes of fNIRS and EEG technologies. Subsequently, we integrated these two methodologies, thereby enhancing the precision and dependability of brain activity monitoring. Following this, we extensively discussed the specific clinical applications of fNIRS-EEG, encompassing ADHD, infantile spasms, depth of anesthesia, IQ estimation, and epilepsy. These investigations underscore the potential clinical utility of the fNIRS-EEG bimodal system, highlighting its significance in neuroscience research and medical diagnostics. Moreover, we aggregated information from 30 papers regarding the fNIRS-EEG bimodal system and its various application scenarios. This highlights the pivotal role of the technique in brain-function research and clinical application. Finally, we scrutinized the challenges confronting fNIRS-EEG dual-modality imaging systems, which encompass hardware and software intricacies, data processing, and standardization. We emphasize that forthcoming trends will revolve around the augmentation of system performance, cost-effectiveness, real-time monitoring capabilities, the refinement of data-fusion and analysis techniques, standardization advocacy, and improved device portability. Vigilant consideration of these trends will empower researchers and clinicians to maximize the potential of the fNIRS-EEG dual-modality imaging system, leading to further advances in neuroscience research and clinical application.

## Data Availability

Not applicable.

## Supplementary Materials

The following supporting information can be downloaded at: https://www.mdpi.com/article/10.3390/brainsci14101022/s1, Tables S1–S3: attachment. References [96,97,98,99,100,101,102,103] are cited in the supplementary materials.
