# Supplementary material for: Strategic Integration: A Cross-Disciplinary Review of the fNIRS-EEG Dual-Modality Imaging System for Delivering Multimodal Neuroimaging to Applications
Source: Brain Sci. 2024 Oct 16;14(10):1022. doi: 10.3390/brainsci14101022 (PMC11506513; doi:10.3390/brainsci14101022)
Supplement: Supplementary file 1 [file brainsci-14-01022-s001.zip › brainsci-3191146-supplementary.pdf]

Table S1 Comparative Analysis of System Attributes

Note: (-) indicates that this information is unknown.

| Time/ References | Number of optical channels | Wavelength (nm) | SDS (mm) | Number of EEG channels | fNIRS/EEG sampling rate (Hz) | Summary |
|---|---|---|---|---|---|---|
| 2016 [78] | 16 | - | 25 | 14 | -/128 | The study employs the EEG-fNIRS framework to uncover a robust correlation between spontaneous facial emotional expression and human brain activity. |
| 2016 [83] | 23 | 695, 830 | 30 | 8 | 10/256 | The study employs the joint independent component analysis method within the EEG-fNIRS system to objectively assess mental stress. |
| 2017 [19] | 52 | 780, 805, 830 | 30 | 64 | 28/1000 | The study introduces a bimodal brain-computer interface system utilizing multiple channels of EEG-fNIRS to achieve a relatively high level of accuracy in distinguishing motion picture tasks. |
| 2017 [82] | 19 | 760, 850 | 30 | 19 | 8.95/250 | The study utilizes EEG-fNIRS to quantify the ability of human mental load. |
| 2017 [75] | 16 | 730, 850 | 25 | 28 | 2/500 | The study employs a fusion of EEG-fNIRS in the decision-making stage to classify three levels of mental workload induced by an n-back working memory task. |
| 2017 [57] | 4 | 690, 830 | 15, 20, 25, 30 | 9 | -/512 | The study delves into the assessment of brain activity during infantile spasms utilizing the EEG-fNIRS system. |
| 2018 [69] | 1 | 670, 850 | - | 2 | 20-80/2000 | The study presents the development of a simultaneous EEG-fNIRS Multimodal System on Chip (SoC) for accurate monitoring of anesthesia depth. |
| 2019 [96] | 12 | - | - | 8 | 7.81/256 | The study introduces a novel hybrid brain-computer interface paradigm that integrates EEG-fNIRS fusion to enhance binary classification accuracy in quantifying the neural correlates of mental arithmetic-induced workload. The study targets two distinct age groups: older and younger individuals, examining the multiscale aspects of cognitive processing. |
| 2019 [49] | 32 | 760, 845 | 30 | 19 | 10/512 | The study employs a bimodal system comprising EEG-fNIRS |

| | | | | | | |
|---|---|---|---|---|---|---|
| | | | | | | for estimating and analyzing the IQ levels of individuals using a regression model. |
| 2019 [29] | 16 | 730, 805, 850 | 25 | 4 | 2/2500 | The study marks the first instance of combining an EEG-fNIRS multi-peak neuroimaging system for the objective diagnosis of ADHD. |
| 2020 [97] | 18 | 760, 850 | - | 13 | 7.81/256 | The study employs an EEG-fNIRS system to record brain activity in patients with Parkinson's disease. Additionally, it captures body movements, including limb movements, and fine movements such as finger movements, using an inertial measurement unit and a WearU glove, respectively. |
| 2020 [86] | 46 | 760, 850 | 30 | 32 | 3.91/500 | The study employs an EEG-fNIRS model to classify subjects into four categories, enhancing the diagnostic and evaluative process for Alzheimer's disease. |
| 2021 [80] | 24 | - | - | 24 | 12.5/4000 | The study enhances the fusion of EEG-fNIRS signals, providing hand-specific interpretability of the encoded forces, which is valuable for motor rehabilitation assessment. |
| 2021 [67] | 128 | 750, 850 | 25-50 | 19 | 20/500 | The study assesses the feasibility of employing the EEG-fNIRS system to investigate cortical hemodynamics associated with status epilepticus, burst suppression, and periodic discharges. This opens new avenues for a better understanding and management of abnormal EEG patterns in neurological ICUs. |
| 2021 [21] | 48 | 695, 830 | - | 19 | 10/256 | The study utilizes EEG-fNIRS to concurrently record hemodynamic responses during ankle movements alongside brain oscillatory activity, laying the foundation for future advancements in brain-computer interfaces for lower extremity rehabilitation. |
| 2021 [98] | 37 | 695, 830 | - | 17 | 10/256 | The study employs the EEG-fNIRS system to assess the impact of low and high noise levels in the workplace on alterations in prefrontal cortex (PFC) activity during stressful psychological tasks. |
| 2021 [72] | 2 | 760, 810, 850 | 30 | 2 | 1/128 | The study introduces a multimodal system for monitoring the depth of anesthesia, utilizing EEG-fNIRS to comprehensively |

| | | | | | | |
|---|---|---|---|---|---|---|
| | | | | | | study both neurological and hemodynamic responses during general anesthesia. |
| 2021[77] | 20 | 690, 830 | 15, 35 | 128 | 10.42/250 | The study assesses the multimodal EEG-fNIRS system as a potent ecological tool for clinically evaluating and early identifying Alzheimer's disease. |
| 2022 [81] | 2 | - | - | 26 | 50/500 | The study scrutinizes the characteristics of the mental load recognition model, refines its signal acquisition configuration, and devises a more precise and user-friendly method for detecting mental load using EEG-fNIRS. |
| 2022 [99] | 36 | - | - | 30 | 10/- | The study introduces a novel framework for multi-level mental workload classification based on EEG-fNIRS features, bolstered by machine learning techniques. |
| 2022 [74] | 37 | 785, 830 | 30 | 36 | 5.21/1200 | The study introduces a comprehensive EEG-fNIRS bimodal signal processing framework for characterizing neural activity elicited by three types of bi-periodic tasks. This framework supports the proposed method as a promising technique for studying neural activity during robot-assisted two-handed training. |
| 2022 [100] | 22 | - | 30 | 28 | 20/1024 | The study introduces a pioneering approach to feature extraction utilizing the EEG-fNIRS system. The study demonstrates that event-related desynchronization and HbO levels during ankle dorsiflexion, along with age, serve as promising biomarkers for stroke motor recovery. |
| 2022 [73] | 36 | - | - | 30 | 12.5/1000 | The study introduces an end-to-end multimodal multitasking neural network model that leverages EEG-fNIRS fusion for decoding various brain physiological signals. |
| 2022 [101] | 44 | - | 30 | 32 | 3.91/500 | The study introduces an integrated multimodal EEG-fNIRS framework, encompassing data collection and analysis, to investigate the impact of personalized music therapy on brain activity as an effective adjunctive therapy. |
| 2023 [102] | 42 | 785, 850 | - | 14 | 3.81/500 | The study investigates the impact of varying light colors on human fatigue levels during simulated driving conditions, along with corresponding alterations in hemodynamics within the visual |

| | | | | | | |
|---|---|---|---|---|---|---|
| | | | | | | and prefrontal cortex. This is achieved through the utilization of an integrated EEG-fNIRS fusion system. |
| 2023 [85] | 24 | - | - | 48 | 10/- | The study introduces a structured sparse multiset canonical correlation analysis method that leverages concurrent recordings from an integrated EEG-fNIRS fusion system. The aim is to discern distinctions in motorized executive, observational, and imagery neural activity involved in cognitive processes. |
| 2023 [84] | 15 | - | - | 28 | 10/500 | The study suggests employing an EEG-fNIRS system for the evaluation of sustained mental fatigue in cockpit applications. |
| 2023 [103] | 8 | - | - | 30 | 10/200 | The study proposes and assesses a Y-shaped neural network, based on the EEG-fNIRS bimodal fusion system, using an open dataset. This network integrates bimodal information at various stages. |
| 2023 [76] | 15 | 760, 850 | 35 | 28 | 10/500 | The study presents an automated diagnosis of depression using feature fusion from EEG-fNIRS data. |
| 2023 [79] | 20 | 762, 845.5 | - | 64 | 50/1000 | The study investigates the synergistic potential of EEG-fNIRS integration in augmenting the decoding accuracy, stability, and fault tolerance of brain-computer interface applications during motor imagery tasks. |

Table S2 fNIRS-EEG dual-modality imaging system, data processing, and analysis

Note: (-) indicates that this information is unknown.

| Time/ References | EEG data preprocessing | fNIRS data preprocessing | EEG feature information extraction | fNIRS feature information extraction |
|---|---|---|---|---|
| 2016 [78] | 30 Hz low-pass filtering, independent component analysis (ICA), artifact removal, baseline correction | 0.1 Hz low-pass filtering, ICA, removal of artifacts, and calculation of concentration changes of HbR, HbO, and Hb using the modified Lambert-Beer law | the logarithm of θ, α, β power spectral densities | mean, median, standard deviation, maximum, minimum, and range of maximum and minimum values for changes in HbO concentration |
| 2016 [83] | 0.5-30 Hz bandpass filtering, ICA, wavelet transform, frequency band division | 0.012- 0.8 Hz band-pass filtering, baseline correction, time-series extraction and moving averages, least-mean linear regression, and | average power density in α, β bands | changes in the average concentration of HbO. |

| | | | | |
|---|---|---|---|---|
| | | calculation of concentration changes of HbR, HbO, and Hb using the modified Lambert-Beer law | | |
| 2017 [19] | 6-30 Hz bandpass filtering, ICA, data window smoothing | 0.02-0.1 Hz bandpass filtering, data segmentation, baseline correction | current source density of electrical signals recorded from the scalp | hurst index fort 10 channels |
| 2017 [82] | 0.5-80 Hz bandpass filtering, 60 Hz trap filtering | 0.01-0.5 Hz band-pass filtering, calculation of concentration changes of HbR, HbO, and Hb using the modified Lambert-Beer law, data segmentation | band power, phase-locked values, phase-amplitude amplification coupling, and left-right hemispheric asymmetrical power | the amplitude of HbO and HbR, slope of HbO and HbR, standard deviation of HbO and HbR, skewness of HbO and HbR, and kurtosis of HbO and HbR |
| 2017 [75] | 1 Hz high pass filtering, 58-62 Hz trap filtering | sliding window motion artifact suppression algorithm, 0.08 Hz low-pass filtering, FIR filter to remove artifacts, calculation of HbR using the modified Lambert-Beer law, concentration change of HbO, data segmentation | power spectral density in the δ (1-4 Hz), θ (4-8 Hz), α (8-13 Hz), β1 (13-20 Hz), and β2 (20-30 Hz) bands | changes in mean activation amplitude relative to baseline for HbO |
| 2017 [57] | 0.5-70 Hz bandpass filtering, a bipolar montage | calculation of HbR and HbO concentration changes using the modified Lambert-Beer law, 0.03-0.5 Hz zero-pHase filter, amplitude normalization, data splitting | power density of θ and δ waves | differences between maximum and minimum HbO concentrations |
| 2018 [69] | - | - | power spectral density | absolute and relative concentrations of HbO, HbR (rHbO = HbO/HbO + Hb,,rHb = Hb/HbO + Hb) |
| 2019 [96] | 0.5-30 Hz bandpass filtering, normalization, data segmentation, wavelet transform, baseline correction | 0.01-0.2 Hz band-pass filtering, calculation of HbR and HbO concentration changes using the modified Lambert-Beer law, data splitting | average of δ, θ, α, β band spectra | integration, slope, maximum, and maximum absolute values of HbO and HbR concentrations |
| 2019 [49] | 0.5-45 Hz bandpass filtering, baseline correction, ICA | elimination of baseline drift, 0.01-0.2 Hz band-pass filtering, calculation of HbO concentration using the modified Lambert-Beer law, calculation of mean values, t-tests | higuchi fractal dimension, Shannon entropy value of wavelet transform coefficients, frequency subband average | slope, mean, variance, kurtosis, skewness, and peak of HbO concentration change |

| | | | | |
|---|---|---|---|---|
| | | | power | |
| 2019 [29] | abnormal data exclusion, 0.05-100 Hz bandpass filter, 45-50 Hz trap filter | exclusion of anomalous saturation data, 0.14 Hz low-pass filtering, calculation of HbR and HbO concentration changes using the modified Lambert-Beer law, data segmentation | lempel-Ziv complexity, fractal dimension, P3 component of ERP signals | changes in concentration of HbO |
| 2020 [97] | ee-trending, 1-99 Hz bandpass filtering | 0.1-0.4 Hz bandpass filtering, the definition of extinction coefficients, and the calculation of HbR and HbO concentration changes using a modified Beer-Lambert law | power density in the θ, α, and β wavebands | changes in concentration of HbO |
| 2020 [86] | 0.5-40 Hz bandpass filtering, 50 Hz trap filtering, ICA, de-artifacts, data segmentation | 0.01-0.2 Hz band-pass filtering, calculation of HbR and HbO concentration changes using modified Beer-Lambert law, data splitting, baseline correction | power densities for θ, δ, β, γ, low-α, and high-α | average changes in HbO and HbR concentrations |
| 2021 [80] | 50 Hz trap filtering, high pass filtering above 0.01 Hz, ICA | 0.25 Hz low-pass filtering, calculation of HbR and HbO concentration changes using a modified Beer-Lambert law | power and phase of θ, α, β, δ, low γ, medium γ and high γ | changes in the concentrations of HbO and HbR |
| 2021 [67] | - | channels with intensities <100 (AU) or signal-to-noise ratios <2 were excluded, raw optical data were converted to optical density, PCA, 0.01-0.2 Hz band-pass filtering, and HbR and HbO concentration changes were calculated using a modified Beer-Lambert law | rhythmic spectrograms, fast Fourier transform spectrograms, asymmetric relative spectrograms, amplitude EEG spectrograms | changes in concentration of HbO |
| 2021 [21] | 0.05-40 Hz bandpass filtering, data segmentation, ICA, wavelet transforms | calculation of HbR and HbO concentration changes using the modified Lambert-Beer law, 0.02-0.4 Hz bandpass filtering, smoothing of the signal, baseline correction, averaging, and normalization | M,ean absolute value, root mean square, waveform length, and fourth-order autoregressive coefficient of the alpha-band power spectrum | variance, kurtosis, and skewness of concentration changes in HbO |
| 2021 [98] | 0.5-70 Hz bandpass filtering, ICA, | calculation of HbR and HbO concentration changes using the modified Lambert-Beer law, 0.012-0.8 Hz band- | α, δ, θ, β band power density | changes in concentration of HbO |

| | | | | |
|---|---|---|---|---|
| | baseline correction, data segmentation, wavelet decomposition | pass filtering, data segmentation, baseline correction | | |
| 2021 [72] | 0.1-45 Hz bandpass filtering, wavelet transform, denoising, de-artifacts | excluding anomalous data, motion artifact correction, response averaging, 0.01-0.10 Hz bandpass filtering, data segmentation | amplitude, spectrum, sample entropy, arrangement entropy\amplitude, spectrum, sample entropy, phase differences between HbO and Hb, power spectral density, average power | average changes in HbO and HbR concentrations |
| 2021[77] | visual inspection, 1-80 Hz bandpass filtering, 50 Hz trap filtering, ICA, wavelet decomposition | raw data conversion to optical density, 0-0.4 Hz band-pass filtering, motion correction algorithm, and calculation of HbR and HbO concentration changes using the modified Lambert-Beer law | average power density in the θ, β, and α bands | standard deviation of changes in HbO and HbR concentrations |
| 2022 [81] | re-referencing, 0.5-45 Hz Bandpass Filter, 50 Hz Trap Filter, ICA, Resampling, Data Splitting | motion artifact removal, 0.5 Hz low-pass filter, data segmentation | power spectral density in the θ, α, β1, and β2 bands | mean, standard deviation, mean square deviation, skewness, root mean square, peak, peak factor, kurtosis, waveform factor, pulse factor, and margin factor for concentration changes of HbO and HbR |
| 2022 [99] | resampling, artifact removal, 1-45 Hz bandpass filtering, data segmentation | calculation of HbR and HbO concentration changes using the modified Lambert-Beer law, 0 - 0.04 Hz bandpass filtering, baseline correction, data partitioning, normalization | power spectral density of θ, α waves | changes in HbO and HbR concentrations |
| 2022 [74] | 1 Hz high-pass filtering, removal of large amplitude pseudo-signals, fast | convert raw fNIRS intensity values to optical density data and rejects undesirable channels, calculates HbR and HbO concentration changes using the modified Lambert-Beer law, corrects for baseline | baseline normalized event-related power spectral density | changes in concentration of HbO |

| | | | | |
|---|---|---|---|---|
| | independent component analysis, 2-36 Hz band-pass filtering, data segmentation | shift and peak artifacts, and 0.05-0.2 Hz bandpass filtering | | |
| 2022 [100] | resampling, 0.05-35 Hz bandpass filtering, ICA, data splitting | calculation of concentration changes of HbR, Hb, and HbO using the modified Lambert-Beer law, 0.01-0.2 Hz band-pass filtering, and data segmentation | power spectral density of task-related events | changes in HbO, Hb, HbR concentrations |
| 2022 [73] | downsampling, baseline removal, 50 Hz power removal, 8-30 Hz bandpass filtering, ICA, channel data filtering, data splitting | downsampling, baseline removal, 0.01-0.2 Hz bandpass filtering, channel data filtering, data partitioning, and calculation of HbR and HbO concentration changes using the modified Lambert-Beer law | feature extraction block is composed of two 2-D convolution layers, a maximum pooling layer, and a batch normalization layer | feature extraction block is composed of a 2-D convolution layer, a maximum pooling layer, and a batch normalization layer |
| 2022 [101] | re-referenced co-averaged references, 0.5-50 Hz bandpass filtering, data segmentation, baseline correction, ICA | 0.01-0.1 Hz band-pass filtering, calculation of HbR and HbO concentration changes using the modified Lambert-Beer law, baseline correction, data splitting | time-domain features (mean, standard deviation, mean of the absolute value of first-order differences, mean of the absolute value of normalized differences, mean of the absolute value of second-order differences, mean of the absolute value of normalized second-order differences), frequency-domain features (power spectral density, Shannon's entropy), time-frequency domain (wavelet entropy, spatial features, ratio of eigenvalues of left-right symmetric electrode pairs) | mean and variance of concentration changes of HbO, Hb |
| 2023 [102] | 1-50 Hz bandpass filtering, ICA, wavelet transform, data segmentation | 0.5 Hz low-pass filtering, calculation of HbR and HbO concentration changes | power spectral density and average power of, θ, α, β, γ waves | changes in HbO and HbR concentration |

| | | | | |
|---|---|---|---|---|
| | | using the modified Lambert-Beer law | | |
| 2023 [85] | 0.3 Hz high-pass filtering, 49 Hz low-pass filtering, removal of artifactual cHannels data, ICA, data segmentation | calculation of HbR, Hb, and HbO concentration changes using the modified Lambert-Beer law, ICA, 0.01 Hz high-pass filtering, 0.5 Hz low-pass filtering, 0.2 Hz band-pass filtering, data segmentation | power density variation in the α-band | changes in HbO and HbR concentrations |
| 2023 [84] | downsampling, 0.5-40 Hz bandpass filtering, 50 Hz trap filtering, motion artifact removal, ICA, data segmentation | downsampling, calculation of HbR and HbO concentration changes using the modified Lambert-Beer law, data segmentation, sequence autocorrelation, correction for motion artifacts, and physiological confounding | variation of the power spectral density of β, α, θ | changes in HbO and HbR concentrations |
| 2023 [103] | common averaging reference, 8-25 Hz bandpass filtering, signal amplitude normalization | calculation of HbR and HbO concentration changes using the modified Lambert-Beer law, 0.01-0.1 Hz bandpass filtering, and baseline removal | mean and slope values of power density variation | changes in HbO and HbR concentrations |
| 2023 [76] | 50Hz Trap Filtering, 0.5-30Hz Band Pass Filtering, Re-referencing, Data Splitting | 0.01-0.1 Hz band-pass filtering, wavelet denoising, and calculation of HbO and HbR concentration changes using the modified Lambert-Beer law | power spectral density in the θ, α bands | concentration changes and sample entropy of HbO |
| 2023 [79] | 4-30 Hz bandpass filtering, data segmentation | 0.02 Hz-0.08 Hz band-pass filtering, calculation of HbR and HbO concentration changes using the modified Lambert-Beer law | variation of signal power density in θ, α, and β bands | changes in HbO and Hb concentrations |

Table 3 Summary of the advantages and disadvantages of the integrated fNIRS-EEG dual-modality imaging system.

| Time/ References | Advantages | Disadvantages |
|---|---|---|
| 2016 [78] | ● demonstrates the utility of a multimodal approach<br>● wireless data transmission | ● fNIRS has a low sampling rate<br>● Ergonomics are less scalable. |
| 2016 [83] | ● an easy way to connect multiple data types<br>● multi-modal advantages are obvious | ● more complex systems<br>● EEG-fNIRS inefficient integration |

| | | |
|---|---|---|
| 2017 [19] | • highly efficient sorting results | • EEG-fNIRS fusion inefficiency<br>• The feature extraction process is cumbersome. |
| 2017 [82] | • extract multiple features for comparative analysis<br>• evaluate the effect of window size on classification performance | • fNIRS only covers the PFC<br>• limited study sample size |
| 2017 [75] | • high efficiency of feature extraction<br>• The advantages of bimodality are obvious. | • memory categorization work study to be studied in depth<br>• low accuracy of classification results |
| 2017 [57] | • Scanning optical changes at different tissue depths uses a multi-distance optical probe.<br>• synchronization of EEG-fNIRS data logging | • sample rate and dynamic range unknown<br>• smaller number of channels |
| 2018 [69] | • microchip-based design, lightweight and compact<br>• wireless operation with shared ADC architecture | • minimal number of channels<br>• poor scalability for large-area monitoring |
| 2019 [96] | • data visualization<br>• better data pre-processing | • fewer channels<br>• few mental testing tasks |
| 2019 [49] | • high number of fNIRS channels<br>• relatively lightweight system | • Wearable portability is in average.<br>• sparse spatial sampling |
| 2019 [29] | • bluetooth module | • a smaller number of channels in the system<br>• limited ergonomic scalability |
| 2020 [97] | • high model accuracy<br>• Selection of the number of features when the highest accuracy is reached by comparison. | • The effect of factors such as age on the results of the experiment was not considered.<br>• small sample size |
| 2020 [86] | • detail data analysis<br>• use different feature sets for different datasets | • Pre-processing of fNIRS data is too simple.<br>• poor ergonomic design |
| 2021 [80] | • compare the classification effects of multiple classifiers<br>• optimize Models with Deep Learning | • The decoding process creates confusion.<br>• poor reliability of decoding capability<br>• complex systems |
| 2021 [67] | • a higher number of channels<br>• analytical methods applicable to long and complex neurological disease data | • Failure to account for factors such as age and gender affects substantive heterogeneity.<br>• Failure to consider systemic physiological factors that also affects cerebral blood flow. |
| 2021 [21] | • experimental tests cover the whole brain<br>• increased sensitivity and specificity of the system | • poor ergonomic scalability<br>• low precision of classification results |
| 2021 [98] | • advantages of multi-frequency band analysis of EEG data<br>• synchronization of EEG-fNIRS data recording | • The experiment covered a small range of head regions.<br>• less wearable portability |
| 2021 [72] | • data visualizations<br>• comparative analysis of multiple features is advantageous | • low number of channels in the system<br>• low acquisition frequency |
| 2021[77] | • The advantages of combining long and | • interference between signals |

| | | |
|---|---|---|
| | short separation channels for measurement are obvious.<br>● whole brain measurement | ● The device is less wearable and portable. |
| 2022 [81] | ● high accuracy of classification results<br>● a wider range of applications | ● EEG acquisition configuration to be optimized<br>● not a real application task scenario |
| 2022 [99] | ● The advantages of five-fold cross-validation technology are obvious.<br>● data visualizations | ● The experimental test range involved only the right frontal region.<br>● presence of volume conduction effects in EEG datasets |
| 2022 [74] | ● EEG-fNIRS data log synchronization<br>● multimodal feedback | ● less wearable portability<br>● limited experimental sample |
| 2022 [100] | ● whole brain measurement<br>● 8-fold cross-validation | ● less wearable portability<br>● limited experimental sample |
| 2022 [73] | ● advantages of multimodal multitasking neural network models<br>● powerful raw data process capabilities and model generalization | ● interference between signals<br>● Other auxiliary tasks have an impact on classification accuracy. |
| 2022 [101] | ● a multimodal integration framework for EEG-fNIRS from data collection to data analysis | ● Excessive amount of features affects classification accuracy.<br>● fNIRS covers a smaller range of brain regions. |
| 2023 [102] | ● multi-group controlled experiments<br>● All electrodes are grounded to a passive electrode. | ● lower fNIRS sampling rate<br>● less portable |
| 2023 [85] | ● Comparative advantages of multitasking are obvious.<br>● EEG-fNIRS data are fused efficiently. | ● less ergonomic scalability<br>● limited experimental sample<br>● high data exclusion rate |
| 2023 [84] | ● use data augmentation to improve model quality<br>● high model performance for bimodal fusion | ● The smaller amount of data severely limits the size of the neural network as well as the final classification performance. |
| 2023 [103] | ● ongoing collection and analysis of fNIRS<br>● whole brain measurement | ● low system sampling rate<br>● The amount of data for the experiment was limited. |
| 2023 [76] | ● whole brain measurement<br>● multi-group controlled experiments | ● EEG-fNIRS feature-level fusion is simpler and ignores the complex relationships among them.<br>● smaller experimental sample size |
| 2023 [79] | ● The advantages of feature fusion methods are more obvious.<br>● separability of data and high reliability of classification results | ● low number of channels<br>● Systematic research is more complex.<br>● limited sample for the experiment |
